# A coupled computational framework for bone fracture healing and long‐term remodelling: Investigating the role of internal fixation on bone fractures

**DOI:** 10.1002/cnm.3609

**Published:** 2022-05-11

**Authors:** Conall Quinn, Alexander Kopp, Ted J. Vaughan

**Affiliations:** ^1^ Biomedical Engineering and Biomechanics Research Centre, School of Engineering National University of Ireland Galway Ireland; ^2^ Meotec GmbH Aachen Germany

**Keywords:** bone fracture healing, finite element modelling, orthopaedic implants

## Abstract

In this study, a coupled computational modelling framework for bone fracture repair is presented that enables predictions of both healing and remodelling phases of the fracture region and is used to investigate the role of an internal fixation plate on the long‐term healing performance of a fracture tibia under a range of different conditions. It was found that introduction of a titanium plate allowed the tibia to undergo successful healing at higher loading conditions and fracture gaps, compared with the non‐plated versions. While these plated cases showed faster rates of repair in the healing phase, their performance was substantially different once they entered the remodelling phase, with substantial regions of stress shielding predicted. This framework is one of the few implementations of both fracture healing and remodelling phases of bone repair and includes several innovative approaches to smoothing, time‐averaging and time incrementation in its implementation, thereby avoiding any unwanted abrupt changes between tissue phenotypes. This provides a better representation of tissue development in the fracture site when compared with fracture healing models alone and provides a suitable platform to investigate the long‐term performance of orthopaedic fixation devices. This would enable the more effective design of permanent fixation devices and optimisation of the spatial and temporal performance of bioabsorbable implants.

## Graphical abstract

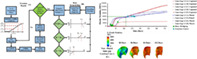

## 1 INTRODUCTION

Secondary bone fracture repair is a four‐stage process^1^ that begins with an inflammatory response and haematoma formation, resulting in granulation tissue formation 3–7 days after the initial fracture. After 2–4 weeks, a fibro‐cartilaginous soft callus is produced from the granulation tissue, which subsequently leads to the development of an ossified hard callus of woven bone surrounding the fracture region after 2–4 months. Over the next several months to years, remodelling of the woven bone takes place to achieve the original pre‐fracture morphology.^1^, ^2^ Assuming the fracture site receives an adequate supply of blood, hormones and growth factors,^3^ fracture healing is largely dictated by the biomechanical conditions in the fracture callus.^4^, ^5^ To enhance the repair process, various types of internal fixation devices are available, whose role is to stabilise the fracture and minimise inter‐fragmentary movement in the early phases of healing. Many complications in bone repair, such as non‐union, implant failure, and delayed healing, are derived from instability in the fracture region.^6^ However, fixation devices themselves can cause late‐stage problems in the repair process, as bone resorption can take place around overly stiff fixators in the remodelling phase, which can increase the likelihood of implant failure.^7^ The ideal fixation device should reduce inter‐fragmentary movement in the early phases of healing to minimise damage to the callus tissue but should enable load‐transfer to the bone itself once the remodelling phase has initiated to avoid unwanted effects of stress shielding.^8^ Understanding the biomechanical performance of these devices represents a challenging mechano‐biological problem that needs to consider in detail the device‐tissue interaction throughout all stages of the bone repair process.

Computational models have been used extensively to understand bone fracture healing, whereby the iterative finite element method has been used to determine the mechanical environment at the site of fracture and local tissue formation proceeds according to appropriate mechano‐regulation theories.^1^, ^2^, ^9^, ^10^ Typically, these models account for a range of biophysical stimuli at the fracture site, including combinations of strain, stress, fluid flow, streaming potentials and acceleration,^11^ that drive biological processes and cellular activities, including migration and proliferation of mesenchymal stem cells (MSCs) from the external environment, their differentiation into fibroblasts, chondrocytes or osteoblasts and tissue formation. While these models require careful calibration of numerical parameters, they have provided a detailed understanding of how local factors, such as mechanical stimuli and cellular activities, affect tissue production following a bone fracture without fixation.^1^, ^2^, ^9^, ^10^ Several studies have also used similar frameworks to explore the role of implant design and fixation conditions on fracture healing response,^12^ with studies that identify reductions in healing time are possible with fixators of increasing stiffness.^12^ However, a limitation of such modelling approaches is that the final remodelling phase of bone repair is rarely fully considered. Instead, these models tend to examine the role of fixation on inter‐fragmentary movement in the early phases of healing, but do not consider wider remodelling processes that take place in both the callus and the adjacent cortex that take place following a successful union over longer time periods.^1^, ^8^, ^12^, ^13^ Healthy bone tissue actively remodels its apparent bone density in response to the local mechanical environment. The constant remodelling of bone from the cellular to organ scales means that bone density is optimised as a function of the loading environment according to Wolff's law. Bone remodelling algorithms are widely used to provide numerical estimates of bone's apparent density due to daily loading according to the theory of mechanostat.^14^ Numerous bone remodelling theories have been proposed, with theories using strain‐derived^15^, ^16^, ^17^ and/or damage‐based^18^, ^19^, ^20^, ^21^, ^22^, ^23^, ^24^ stimuli to describe cellular activity^23^, ^25^ and drive the remodelling process. While these theories support the concept that bone remodelling is a targeted mechanism to simultaneously preserve bone mass and prevent fractures, bone remodelling also plays a vital role in the final stage of the fracture repair process, as woven bone is gradually replaced with orientated lamellar bone to return to original pre‐fracture morphology. However, these remodelling processes have rarely been considered in existing healing algorithms, which means that the longer‐term tissue responses have not been fully explored. The ability to predict the response of bone tissue to adaptations in mechanical loading^26^ is of vital significance when considering how the introduction of load‐bearing implant will affect the structural architecture. Identifying a fixation device that has optimal stability in the early phases fracture repair and an appropriate design to minimise stress shielding requires computational models of fracture repair that consider both fracture healing and subsequent remodelling responses. These approaches could be particularly relevant given the ongoing development of bioabsorbable fixation devices, which provide the potential to control both spatial and temporal load‐bearing performance during healing.

The objective of this study is to develop a coupled modelling framework for bone fracture repair that predicts both fracture healing and remodelling phases of the tissue over a period of 12 months. During the fracture healing phase, the model predicts tissue production based on a combination of the biophysical stimuli and cellular concentration in the fracture callus. The remodelling phase is only initiated once a suitable fracture union is achieved and considers both damage and strain‐energy density (SED) based tissue remodelling. The coupled modelling framework for bone fracture repair is used to investigate the role of an internal fixation plate on the long‐term healing performance under a range of loading conditions.

## 2 MATERIALS AND METHODS

### 2.1 Model geometry

**FIGURE 1:**
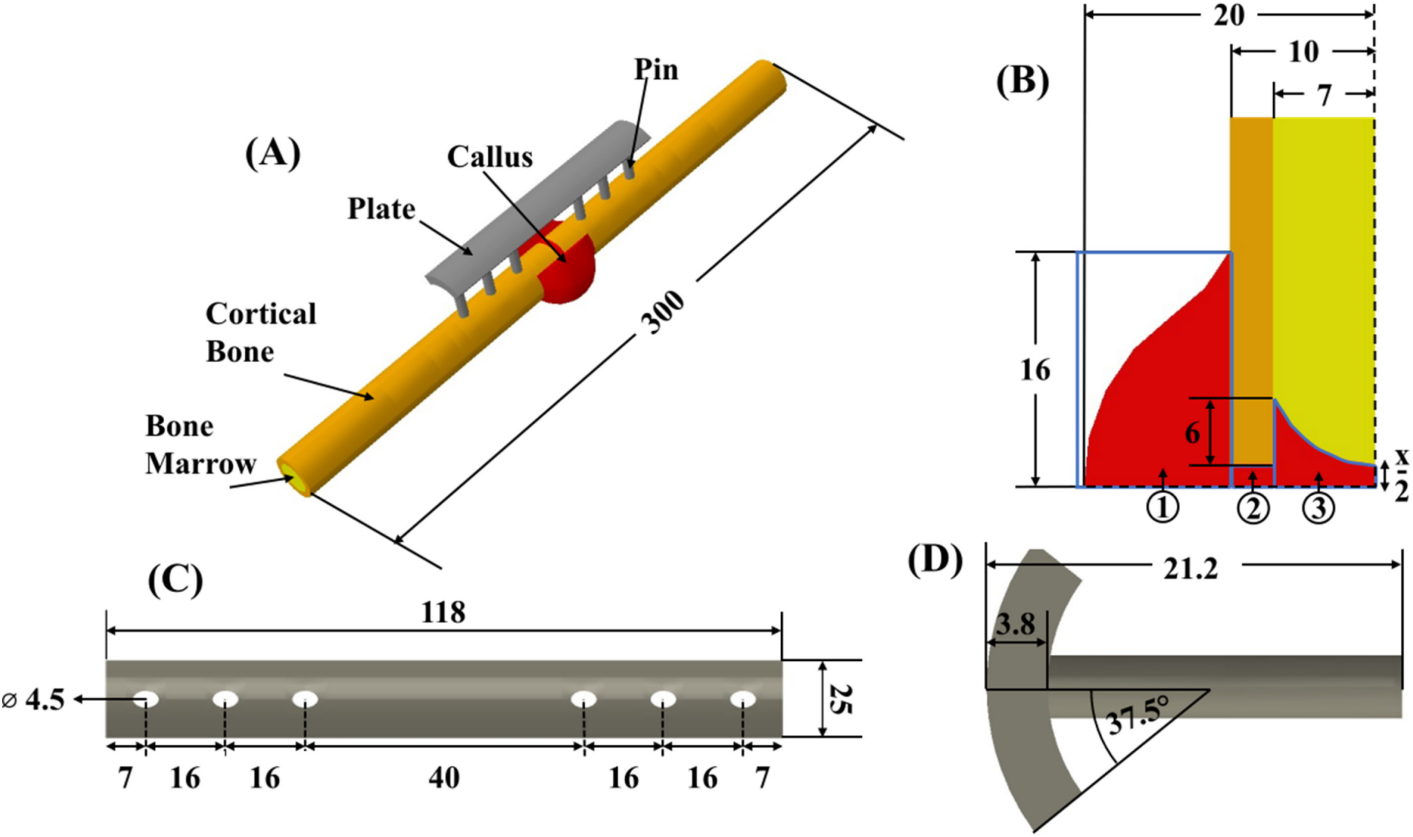
Geometric information on human tibia implanted and external fixator; (A) bone plate; (B) callus regions; (C) top‐down view of plate without pins; (D) side view of plate with pins. All measurements were recorded in millimetres

Three‐dimensional finite element models of non‐plated and plated long‐bone tibia fractures were developed, according to dimensions provided in Figure 1. The tibia was simplified as a smooth co‐axial cylinder composed of cortical bone and bone marrow at the centre (see Figure 1A), with the shape and dimensions based on previous studies.^9^, ^27^, ^28^ The fracture callus was divided into three zones, (1) outer callus, (2) callus focus and (3) inner callus. Several fracture gaps (*x* = 0.75, 2.5, 5, 8 and 12 mm) were considered. For the plated models, six self‐locking screws fastened a titanium bone plate to the tibia^29^ (see Figure 1C,D). The cortical bone, callus, bone marrow and screw‐plate system were modelled with 32,076, 17,750 and 15,710 8‐noded linear brick, trilinear displacement, pore pressure and temperature, reduced integration elements (C3D8RPT).

### 2.2 Bone repair model

#### 2.2.1 General overview

**FIGURE 2:**
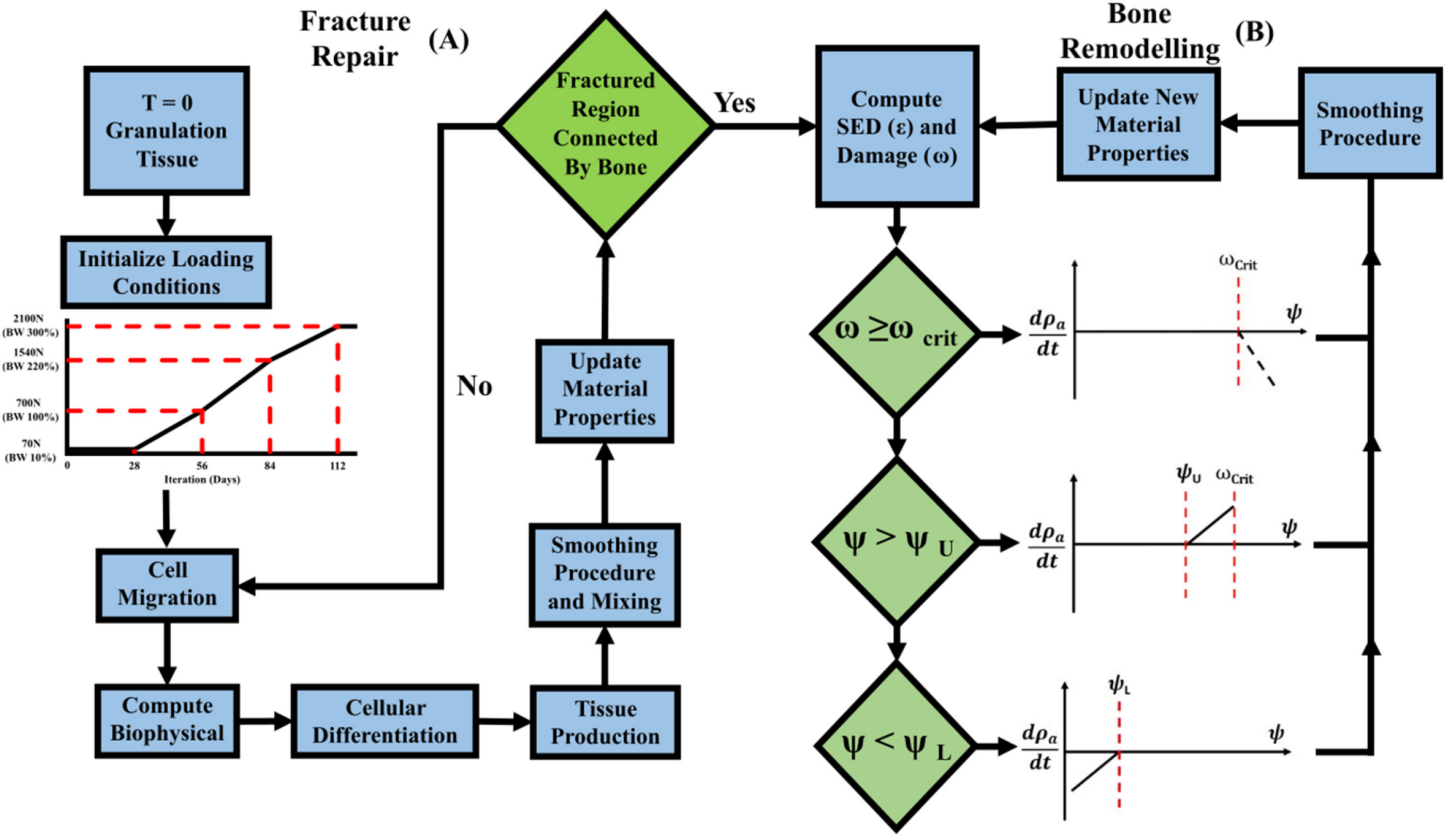
Iterative calculations for tissue development for bone fracture repair and bone remodelling; (A) Bone fracture repair algorithm and (B) SED/damage‐based bone remodelling

The bone repair model is represented schematically in Figure 2, whereby an iterative approach that considers both (a) fracture healing and (b) remodelling phases of bone repair was developed. For the fracture healing component (see Figure 2A), a fracture callus comprised of granulation tissue is infiltrated by considering angiogenesis and migration of MSCs from the adjacent undamaged cortical ends (see Section 2.2.1). As the patient gradually loads the fractured bone, biophysical stimuli acting on the cells drive differentiation and tissue production process, with gradual changes from one phenotype to another enabled by a smoothing process within the fracture healing algorithm (See Section 2.2.3). Once adequate woven bone has formed in the “callus focus” region (see Figure 2B), the entire fracture callus automatically enters the bone remodelling algorithm, whereby both damage and SED based remodelling is predicted. The remodelling algorithm updates the bone material properties according to one of four processes; (1) damage‐based remodelling; (2) positive SED remodelling; (3) negative SED remodelling; (4) no remodelling (See Section 2.2.4). The apparent bone density continuously remodels to achieve the homeostatic bone energy density. The model is implemented in the Abaqus/Standard finite element framework through a series of user‐defined field Subroutines (USDFLD).

#### 2.2.2 Blood vessel growth/cell migration and proliferation

**FIGURE 3:**
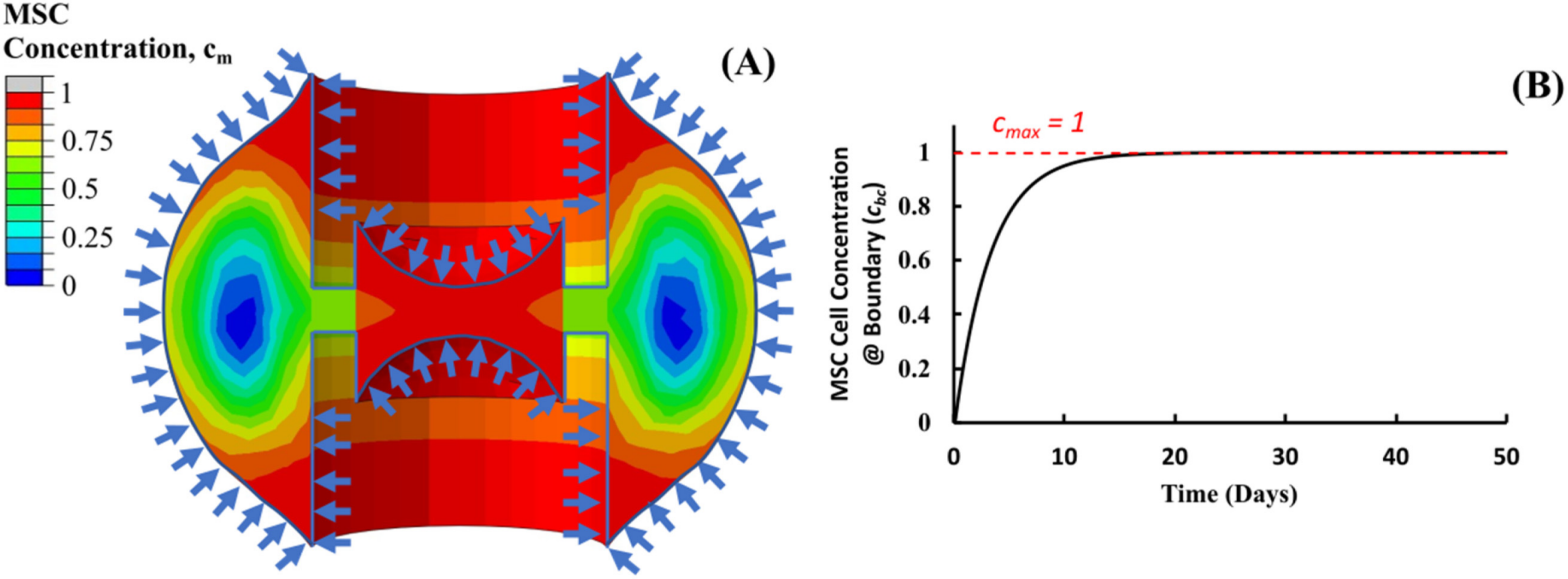
Overview of diffusion model used to describe the infiltration of MSCs/development of blood vessels; (A) MSCs invading the fracture callus from the surrounding soft tissue, bone marrow and inner cambial of the periosteum; (B) Cellular concentration magnitude applied to surface shown in (A)

A diffusion model was implemented in the fracture callus to model the infiltration of MSCs and the development of blood vessels according to,

(1) $$\frac{dc_{m}}{\mathrm{dt}}=D\nabla^{2}c_{m}$$

where $C_{m}$ is the cell concentration of MSCs, *D* is the mass diffusion coefficient. This diffusion model was implemented in Abaqus using a thermal analogy, given the similarity between the thermal diffusion and mass diffusion (Fick's second law) equation. This approach has been applied by other studies, with further details of the implementation process available in Reference 2. Briefly, the diffusion model assumes that the movement of the cells is random and non‐directional, with the local relative cellular concentration ($c_{\text{cell}})$ ranging from $0\leq c_{\text{cell}}\leq 1$. The source of the progenitor cells was the surrounding soft tissue, bone marrow and inner cambial of the periosteum (see Figure 3A).^30^ At the beginning of simulations (t=0), the callus does not contain any MSCs $\left(c_{m}=0\right)$ and consists of only granulation tissue. The cell concentration $c_{m}$ changes with respect to time according to Equation (1). The diffusion coefficient was determined a hit and trail method implemented by previous studies^23^, ^24^, ^25^ whereby a steady state cell concentration was achieved after 112 days. Diffusion coefficient of *D =* 0.1 mm^3^/day yielding the best results for cellular confluence following 112 days. The diffusion model was used to describe the migration of all cells under the assumption that cell movements were random and non‐directional. Cell concentration at the integration points were calculated through interpolating nodal cell concentrations. Previous studies^2^, ^31^ applied the maximum cellular concentration $\left(c_{m}=1,T_{\text{nodal}}=1,@t=0\right)$ at the boundaries shown in Figure 3A. However, this results in the integration points of these elements having a cellular concentration equal to half of the applied cellular concentration at time zero $\left(c_{m}=0.5,@t=0\right)$, and causes these elements to stiffen much quicker than the rest of the elements within the fracture callus. Therefore, using Equation (2), a temporal non‐linear magnitude was created (Figure 3B) and applied to the surface of the fracture callus highlighted within Figure 3A.

(2) $$\;c_{\mathrm{bc}\left(t+1\right)}=c_{\mathrm{bc}\left(t\right)}+D\left(c_{\max}-c_{\mathrm{bc}\left(t\right)}\right)*\left(\mathrm{dt}\right)$$

#### 2.2.3 Fracture healing mechano‐regulation algorithm

**FIGURE 4:**
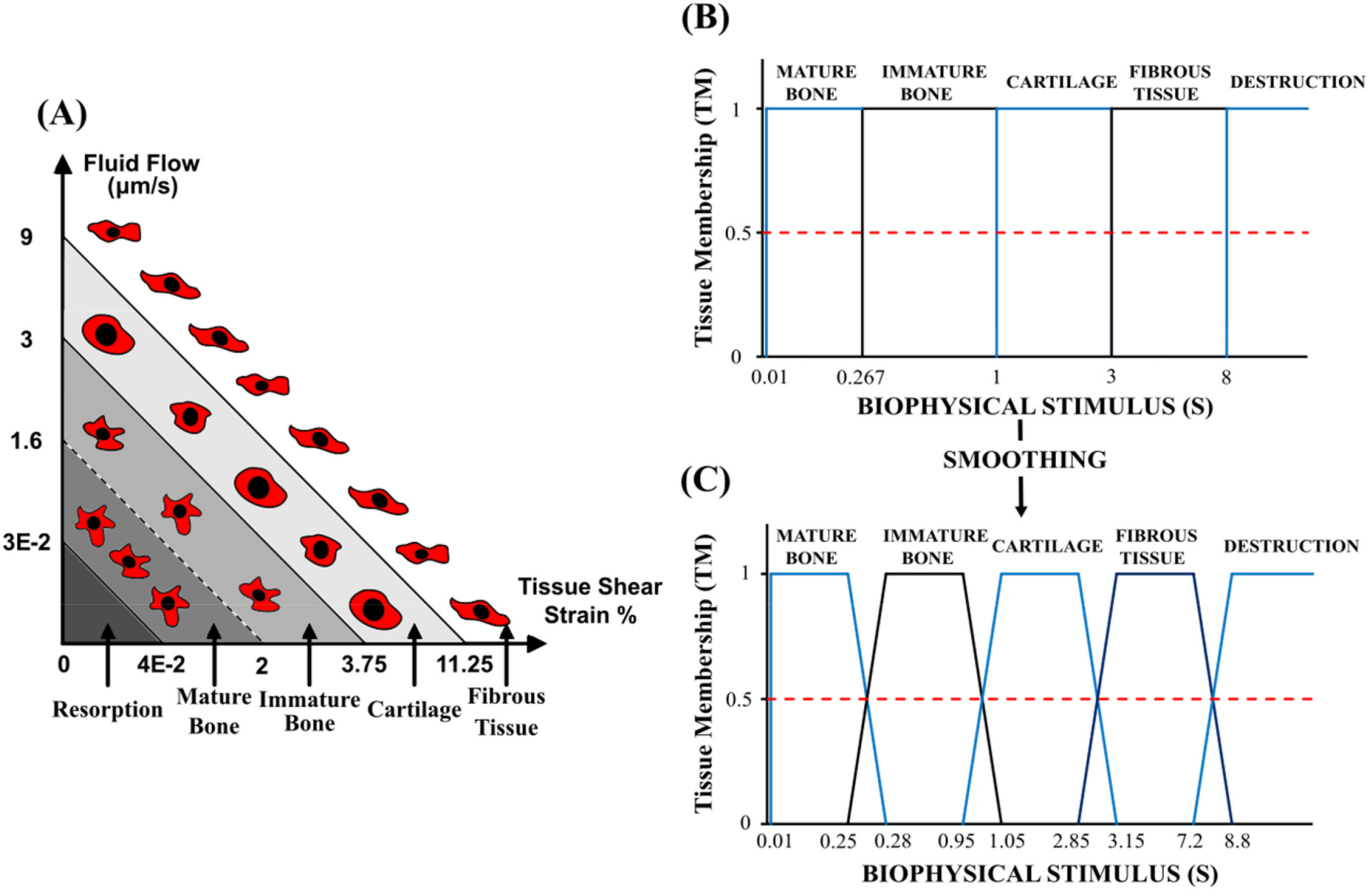
(A) The mechano‐regulation model based on deviatoric strain and fluid velocity. (B) Smoothed mechanobiological regulation based on Sapotnick et al.^34^

The biphasic mechano‐regulation theory described by Prendergast^32^ was used to govern MSC differentiation and tissue phenotype under mechanical loading within the callus tissue (see Figure 4A). It was assumed that the tissue was biphasic, and completely saturated, such that the sum of the volume fractions of the solid ($V^{s}$) and fluid ($V^{f}$) phase must equal one.

(3) $$V^{s}+V^{f}=1$$

Mechanical loading produced a deviatoric strain (shear deformation) and a fluid flow throughout the fracture callus. The effective pore fluid velocity ($w_{f}$) was calculated as the velocity of the fluid phase $\left(v_{f}\right)$ with respect to the solid phase $\left(v_{s}\right)$.

(4) $$w_{f}=v_{f}-v_{s}$$

The fluid flow velocity $\left(w_{\mathrm{mag}}^{f}\right)$ was calculated using Equation (5). The deviatoric strain $\;\left(\varepsilon_{\mathrm{ds}}^{s}\right)$ was calculated using Equation (6), which considers this term as a function of the principal strains (*ε*_*1*_ *, ε*_*2*_ *, ε*_*3*_). The local elemental stimulus (*S*) (Equation (7)) taken directly from biphasic mechano‐regulation theory,^21^ which was defined as the sum of the equivalent deviatoric strain and the interstitial fluid velocity in which a=0.0375 and b=3μm/s. ^33^

(5) $$w_{\mathrm{mag}}^{f}=\sqrt{w^{f}.w^{f}}$$

(6) $$\varepsilon_{\mathrm{ds}}^{s}=\frac{2}{3}\sqrt{{\left(\varepsilon_{1}-\varepsilon_{2}\right)}^{2}+{\left(\varepsilon_{2}-\varepsilon_{3}\right)}^{2}+{\left(\varepsilon_{3}-\varepsilon_{1}\right)}^{2}}$$

(7) $$S=\frac{\left(\varepsilon_{\mathrm{ds}}^{s}\right)}{a}+\frac{w_{\mathrm{mag}}^{f}}{b}$$

The local stimulus within an element controlled whether the MSCs within that element differentiated into fibroblasts, chondrocytes or osteoblasts. As a rule, high levels of mechanical stimuli $\;\left(S>3\right)$ resulted in the formation of fibrous tissue, intermediate levels of stimulation $\;\left(1<S<3\right)$ promoted cartilaginous tissue, lower levels of stimulus resulted in formation of immature bone $\left(0.2667<S<1\right)$, while woven bone was formed between when the stimulus was 0.01<S<0.2667. For very low stimuli $\left(0.01<S\right)$ callus resorption occurs, whereby osteoclasts removed non‐loaded bone tissue.^2^ These mechano‐regulation rules were implemented considering fuzzy zones,^34^ that allowed for gradual changes in tissue properties as differentiation occurred, whereby two tissue phenotypes could be produced simultaneously, allowing for smoother transitions between cell phenotypes. Sapotnick and Nackenhorst altered the range of their fuzzy zones from 50% to 400% with minimal effects on healing outcomes. A relatively conservative approach was taken when determining the width of the fuzzy zones, whereby the range was determined by taking 10% of the threshold stimulus $\left(S=3,S=1,S=0.2667,S=0.01\right)$.

Tissue membership $\left(\mathrm{TM}\right)$ was determined by the biophysical stimulus (Figure 4A). The tissue membership level‐controlled cell differentiation and tissue production. The local TM for fibrous, cartilage, immature bone and mature bone ranged from $0\leq {\mathrm{TM}}_{f,c,\mathrm{im},\mathrm{mb}}\leq 1$. Maximum tissue membership (TM=1) of a phenotype resulted in complete recruitment of available cells, while zero tissue membership (TM=0) resulted in no recruitment. The evolution of fibroblasts, chondrocytes, immature bone cells and mature cells and their associated tissues are described by Equations ((8), (9), (10), (11)).^35^ Here $c_{m},c_{f},c_{c},c_{\mathrm{ib}}$ and $c_{\mathrm{mb}}$ are normalised mesenchymal, fibroblast, chondrocyte, immature bone, and mature bone cell densities, respectively, $F_{f},F_{c},F_{\mathrm{ib}}\;$ and $F_{\mathrm{mb}}$ are cell differentiation rates. The differential potential of MSCs, fibroblasts, chondrocytes, immature bone cells and mature cells was ordered from highest to lowest respectively. Cells were allowed to differentiate into different cell phenotypes of lower differential potential. As shown in Equation (9), given ${\mathrm{TM}}_{c}>0$, MSC ($c_{m})$ and fibroblasts $\left(c_{f}\right)$ differentiated into chondrocytes. However, if ${\mathrm{TM}}_{\mathrm{ib}}>0$ or ${\mathrm{TM}}_{\mathrm{mb}}>0$, chondrocytes were differentiated immature or mature bone cells, respectively.

(8) $$\frac{{\mathrm{dc}}_{f}}{\mathrm{dt}}=\left(F_{f}*{\mathrm{TM}}_{f}\right)\;\left(1-c_{f}\right)c_{m}-\left(F_{c}*{\mathrm{TM}}_{c}\right)\left(1-c_{c}\right)c_{f}-\left(F_{\mathrm{ib}}*{\mathrm{TM}}_{\mathrm{ib}}\right)\left(1-c_{\mathrm{ib}}\right)c_{f}-\left(F_{mb}*{\mathrm{TM}}_{\mathrm{mb}}\right)\left(1-c_{\mathrm{mb}}\right)c_{f})$$

(9) $$\frac{{\mathrm{dc}}_{c}}{\mathrm{dt}}=\left(F_{c}*{\mathrm{TM}}_{c}\right)\left(1-c_{c}\right)\left(c_{m}+c_{f}\right)-\left(F_{\mathrm{ib}}*{\mathrm{TM}}_{\mathrm{ib}}\right)\left(1-c_{\mathrm{ib}}\right)c_{c}-\left(F_{\mathrm{mb}}*{\mathrm{TM}}_{\mathrm{mb}}\right)\left(1-c_{\mathrm{mb}}\right)c_{c})$$

(10) $$\frac{{\mathrm{dc}}_{\mathrm{im}}}{\mathrm{dt}}=\left(F_{\mathrm{im}}*{\mathrm{TM}}_{\mathrm{im}}\right)\left(1-c_{\mathrm{im}}\right)\left(c_{m}+c_{f}+c_{c}\right)-\left(F_{\mathrm{mb}}*{\mathrm{TM}}_{\mathrm{mb}}\right)\left(1-c_{\mathrm{mb}}\right)c_{\mathrm{im}}$$

(11) $$\frac{{\mathrm{dc}}_{\mathrm{mb}}}{\mathrm{dt}}=\left(F_{\mathrm{mb}}*{\mathrm{TM}}_{\mathrm{mb}}\right)\left(1-c_{\mathrm{mb}}\right)\left(c_{m}+c_{f}+c_{c}+c_{\mathrm{im}}\right)\;$$

Tissue production and replacement is regulated by the corresponding cells, tissues themselves and TM. The volume fraction of fibrous tissue ($m_{f})$, cartilage ($m_{c})$, immature bone ($m_{\mathrm{ib}})$, and mature bone ($m_{\mathrm{mb}})$ are described by (Equations ((12), (13), (14), (15))). Granulation tissue, fibrous tissue, cartilage, immature bone and mature bone were given priority in order, respectively. Therefore, allowing cartilaginous tissue to replace fibrous tissue within the fracture callus, however cartilaginous tissue cannot replace immature or mature bone (see Equation (14)). Initially $\left(T=0\right)$ the entire fracture callus consists of granulation tissue ($m_{\text{gran}}=1)$. Granulation tissue was replaced by according to Equation (16).

(12) $$\frac{{\mathrm{dm}}_{\mathrm{mb}}}{\mathrm{dt}}=\left(Q_{\mathrm{mb}}*{\mathrm{TM}}_{\mathrm{mb}}\right)\left(1-m_{\mathrm{mb}}\right)\left(c_{\mathrm{mb}}\right)\;$$

(13) $$\frac{{\mathrm{dm}}_{\mathrm{ib}}}{\mathrm{dt}}=\left(Q_{\mathrm{ib}}*{\mathrm{TM}}_{\mathrm{ib}}\right)\left(1-m_{\mathrm{ib}}-m_{\mathrm{mb}}\right)\left(c_{\mathrm{ib}}\right)-{\mathrm{TM}}_{\mathrm{mb}}Q_{\mathrm{mb}}c_{\mathrm{mb}}m_{\mathrm{ib}}m_{\mathrm{tot}}$$

(14) $$\frac{{\mathrm{dm}}_{c}}{\mathrm{dt}}=\left(Q_{c}*{\mathrm{TM}}_{c}\right)\left(1-m_{c}-m_{\mathrm{ib}}-m_{\mathrm{mb}}\right)\left(c_{c}\right)-\left({\mathrm{TM}}_{\mathrm{mb}}Q_{\mathrm{mb}}c_{\mathrm{mb}}-{\mathrm{TM}}_{\mathrm{ib}}Q_{\mathrm{ib}}c_{\mathrm{ib}}\right)m_{c}m_{\mathrm{tot}}$$

(15) $$\frac{{\mathrm{dm}}_{f}}{dt}=\left(Q_{f}*{\mathrm{TM}}_{f}\right)\left(1-m_{f}-m_{c}-m_{\mathrm{ib}}-m_{\mathrm{mb}}\right)\left(c_{f}\right)-\left({\mathrm{TM}}_{\mathrm{mb}}Q_{\mathrm{mb}}c_{\mathrm{mb}}-{\mathrm{TM}}_{\mathrm{ib}}Q_{\mathrm{ib}}c_{\mathrm{ib}}-{\mathrm{TM}}_{c}Q_{c}c_{c}\right)m_{f}m_{\mathrm{tot}}$$

(16) $$m_{\text{gran}}=1-m_{f}+m_{c}+m_{\mathrm{ib}}+m_{\mathrm{mb}}$$

A time control $\left({\mathrm{\Delta t}}_{\text{fracture}}\right)$ within the fracture healing algorithm was implemented to ensure the change in tissue volume fraction did not exceed the maximum threshold value $\left(\Delta m_{\max}=0.05\right)$ for any given time increment. If $\left({\mathrm{dm}}_{\mathrm{mb},}dm_{\mathrm{ib}},dm_{c},dm_{\mathrm{mf}}>\Delta m_{\max}\right)$ the time increment was updated according to Equation (17), ensuring model accuracy regarding bone fracture repair was maintained.

(17) $${\mathrm{\Delta t}}_{\text{fracture}}=\frac{\Delta m_{\max}}{\max\left({\mathrm{dm}}_{\mathrm{mb}},{\mathrm{dm}}_{\mathrm{ib}},{\mathrm{dm}}_{c},{\mathrm{dm}}_{f}\right)},dt_{\mathrm{new}}={\mathrm{\Delta t}}_{\text{fracture}}*dt_{\mathrm{old}}$$

The rule of mixtures (RoM) was used to calculate the predicted elastic modulus of the elements within the fracture callus for each increment using Equation (18). The RoM calculated the predicted elastic modulus before smoothing occurred,

(18) $$E_{\text{fracture}}=\left(\left(m_{\text{gran}}\right)*\left(E_{\text{gran}}\right)+\left(m_{f}\right)*\left(E_{f}\right)+\left(m_{c}\right)*\left(E_{c}\right)+\left(m_{\mathrm{ib}}\right)*\left(E_{\mathrm{ib}}\right)+\left(m_{\mathrm{mb}}\right)*\left(E_{\mathrm{mb}}\right)\right)$$

The implementation of this mechano‐regulation algorithm also included a further smoothing strategy, whereby the elastic modulus ($E_{n+1})$ was calculated by obtaining a time averaged value of the elastic modulus from the previous 10 days according to,

(19) $$E_{t+\mathrm{dt}}=\frac{1}{10}\sum_{t=t_{i}-10}^{t=t_{i}}\left(E_{t}\right)*\left(dt_{t}\right)$$

Many previous studies have implemented time averaging using a fixed increment size.^2^, ^35^, ^36^ However, using a variable time step with custom time controls allows for greater accuracy and versatility as the time step $\left({\mathrm{\Delta t}}_{\text{fracture}}\right)\;$ is controlled by changes in cellular concentration and prevents abrupt changes in cell/tissue concentrations. Fracture healing continued within the callus until the “callus focus” region was connected by differentiated woven bone and the change in elastic modulus due to SED surpassed the change in elastic modulus due bone fracture repair. Following this, the fracture callus underwent bone remodelling whereby SED/micro‐damage were the controlling stimuli.

#### 2.2.4 Coupled strain/damage‐based remodelling

**FIGURE 5:**
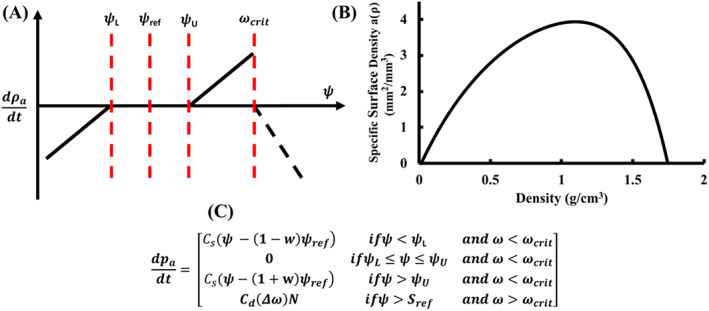
(A) Bone remodelling algorithm using SED as the mechanical stimulus for the implant; (B) Surface area density of bone $\left(a\left(\rho \right)\right)$ as a function of bone density^41^

The coupled strain‐ and damage‐adaptive algorithm outlined by McNamara and Prendergast^21^ was used to simulate bone remodelling. Strain‐based remodelling used SED as the remodelling stimulus $\left(\psi =U/\rho \right)$, where U is the strain energy, ρ is the apparent bone density. A site‐specific approach was implemented whereby each bone element tried to reach a homeostatic state of SED. The homeostatic SED reference point $\left(\psi_{\mathrm{Ref}}\right)$ is calculated according to Equation (20) (see Figure 5A).

(20) $$\psi_{\mathrm{Ref}}=\frac{\sigma_{U}^{2}}{\left(2\right)\left(E_{U}\right)\left(\rho_{U}\right)}$$

Where $\sigma_{U},E_{U,}\;\rho_{U}$ are the homeostatic values for stress, elastic modulus and density, which have values of 6.6 MPa, 17,000 MPa and 1.649 g/cm^3^, respectively. This however creates can cause difficulties as it creates a dependency between the final converged solution and the initial state and width of lazy zone $\left(w\right).$ ^37^ Bone elements remodel towards the upper $\left(\psi_{U}\right)$ and lower $\left(\psi_{L}\right)$ SED values, therefore the formulation $\psi_{\mathrm{Ref}}$ was modified (see Equation (21)) within this paper in order to allow bone to positively remodel to its homeostatic elastic modulus under normal loading conditions.

(21) $$\psi_{\mathrm{Ref}}=\frac{\sigma_{U}^{2}}{\left(2\right)\left(E_{U}\right)\left(\rho_{U}\right)}*\frac{1}{\left(1+\text{width}\right)}$$

The upper and lower reference point for SED was were described by Equations (22) and (23). Within this modified approach altering the value of w only altered the positions of $\psi_{\mathrm{Ref}}$ and $\;\psi_{L}$, while $\;\psi_{u}$ remains constant. The region of reabsorption due to SED could be increased by decreasing w, while increasing w resulted in the opposite effect.

(22) $$\;\psi_{u}=\left(\psi_{\mathrm{Ref}}\right)*\left(1+w\right)$$

(23) $$\;\psi_{L}=\left(\psi_{\mathrm{Ref}}\right)*\left(1-w\right)$$

The width $\left(w\right)$ value controlled the size of the lazy zone, and the degree of reabsorption. The width $\left(w\right)$ had no effect on the value of $\psi_{U}$ as it has no dependency. In line with previous studies, a width of 0.35 was chosen.^38^ Bone remodelling was simulated as a change in apparent bone density. Carter and Hayes found that the elastic modulus of cortical and trabecular bone is approximately proportional to the cube and square respectively of the apparent bone density,^39^ this relationship between density (g/cm^3^) and elastic modulus (MPa) is described by,

(24) $$E=3790\rho^{3}$$

The rate of remodelling is affected by the amount of free surface area, as remodelling occurs at the pore surfaces. Martin^40^ determined the internal free surface area as a function of the apparent density $A\left(\rho \right)$. The internal free surface area per unit volume $a\left(\rho \right)=A\left(\rho \right)/V$ is shown in Figure 5B. At maximum bone density $\rho_{\max}=1.73g/{\mathrm{cm}}^{3}$ and the minimum bone density $\rho_{\min}=0.01g/{\mathrm{cm}}^{3},$ no bone remodelling occurs, as the free surface area equals zero. The remodelling rules and formulae that determine the rate of remodelling are shown in Figure 5C.

A strain remodelling rate was determined from $C_{s}=a\left(\rho \right)\tau$, where τ is a time constant with a value of $\tau =130g^{2}mm^{-2}J^{-1}$ per month, and $a\left(\rho \right)$ is the specific surface area.^42^ Time controlled incrementation $\left({\mathrm{\Delta t}}_{\mathrm{SED}}\right)$ was implemented such that for any given iteration the maximum allowable density change $\left(\Delta \rho_{\max}\right)$ was $0.175g/{\mathrm{cm}}^{3}$ per month. If the density change for any given iteration exceeded the allowable maximum, then time increment $\left(dt_{\mathrm{new}}\right)$ was reduced according to,

(25) $${\mathrm{\Delta t}}_{\mathrm{SED}}=\frac{\Delta \rho_{\max}}{\left(a\left(\rho \right)*\tau *\left(S-S_{U,L}\right)*\mathrm{dt}\right)},dt_{\mathrm{new}}={\mathrm{\Delta t}}_{\mathrm{SED}}*dt_{\mathrm{old}}$$

Damaged bone tissue within bone is constantly removed and replaced with healthy bone tissue. At equilibrium, damage $\left(\omega_{\mathrm{RE}}\right)$ exists within bone due to the cyclic loading of bone tissue. Damage is calculated within each element using the remaining life approach, whereby undamaged bone is denoted by ω=0, while fracture bone is denoted by ω=1. ^43^ It is assumed within healthy bone tissue that damaged is removed at a constant rate which is denoted by ${\dot{\omega }}_{\mathrm{RE}}$. If the damage formation rate $\dot{\omega }$ exceeds the repair rate ${\dot{\omega }}_{\mathrm{RE}}$ damage is accumulated within the bone tissue it will create damage remodelling stimulus Δω as shown in Equation (26).

(26) $$\Delta \omega =\int_{0}^{t}\left(\dot{\omega }-{\dot{\omega }}_{\mathrm{RE}}\right)\mathrm{dt}$$

The damage formation rate was assumed to be linear and was implemented using Miner's rule,^44^ which determined the damage formation rate $\dot{\omega }$ and repair rate ${\dot{\omega }}_{\mathrm{RE}}$. Previous experimental data by Carter et al.^45^ provides the number of cycles to failure ($N_{f}$) for several different stress levels. Using Equations (27) and (28), the damage and repair rates were calculated, where σ is the stress in MPa, T the temperature (37°C) and H, J, T and M are empirical constants.

(27) $$\dot{\omega }=\frac{1}{N_{f}}$$

(28) $$\log\left(N_{f}\right)=\text{Hlog}\left(\sigma \right)+\mathrm{JT}+\mathrm{K\rho}+M$$

Once the accumulated damage exceeds a critical damage threshold ($\omega_{\text{crit}})$, damage‐based resorption occurred. The critical damage level was determined at 3500 μɛ, with $\;\psi_{U}$ located at 1500 μɛ. Therefore, the critical damage threshold is situated 2000 μɛ above $\;\psi_{u}$ or ~1.3 times the strain of $\;\psi_{U}$. Damage can exist within the bone without activating damage‐based reabsorption, until the damage critical $\omega_{\text{crit}}\;$ damage threshold is exceeded. Similarly, to the SED‐based remodelling a damage‐based time control (${\mathrm{\Delta t}}_{\text{damage}})$ was implemented (Equation (29)). A damage rate constant of $C_{d}=0.1e^{8}$ was used.^42^ Simulations considered cases with damage (D) and without damage **(**ND) based remodelling for the plated models

(29) $${\mathrm{\Delta t}}_{\text{damage}}=\frac{0.00625}{\left(a\left(\rho \right)*C_{d}*N*\omega \right)},dt_{\mathrm{new}}={\mathrm{\Delta t}}_{\text{damage}}*dt_{\mathrm{old}}$$

Within combined fracture healing/remodelling algorithm the smallest value of ${\mathrm{\Delta t}}_{\text{fracture}},{\mathrm{\Delta t}}_{\mathrm{SED}}\;\text{and}\;{\mathrm{\Delta t}}_{\text{damage}}$ was always implemented in order to determine the new time increment $dt_{\mathrm{new}}$.

### 2.3 Boundary conditions

**FIGURE 6:**
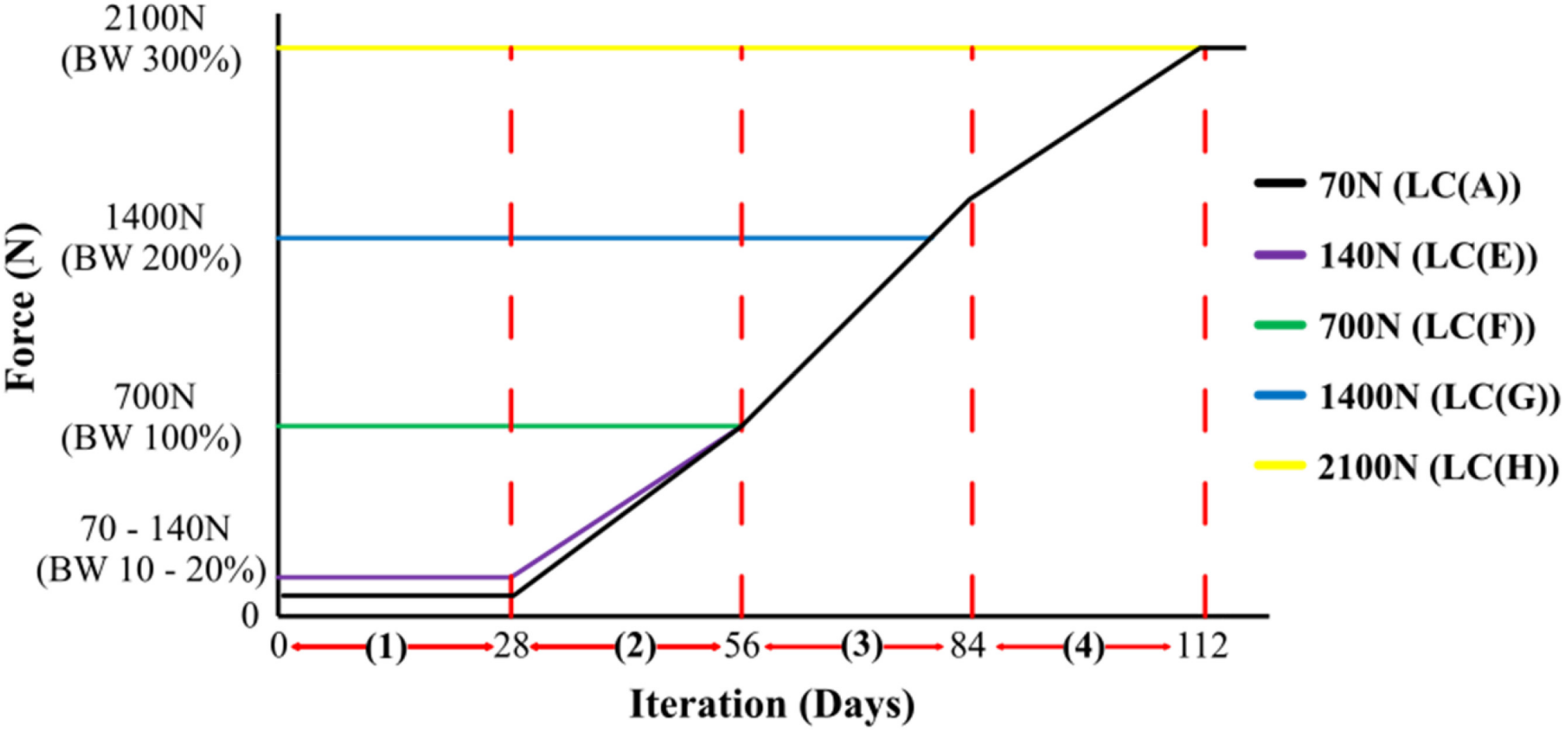
Post‐operative loading conditions

Post‐operative loading of the tibia contained four different loading phases **(**1) indirect loading, (2) walking with crutches, (3) imperfect gait and (4) perfect gait (See Figure 6).^36^ The body weight of a healthy patient was assumed to be 70 kg. All loading conditions were applied as scaled uniaxial compression. An initial load of 10% of body weight (BW) was applied for the first 28 days in order to simulate indirect loading. From days 28 to 56 this tibia was loaded to simulate the patient walking with crutches, gradually transferring more load through the injured tibia. The load transferred through the tibia continually increased between days 56–112 as the patient walked with an imperfect gait. Following 112 days, a normal gait of 300% BW was implemented. The initial loading conditions (LC) were altered between 0 and 112 days, according to several different conditions described by LC(A)–LC(H), as shown in Figure 6. LC(B)–LC(D) exist between LC(A) and LC(E) and have an initial load of 87.5, 105, 122.5 N. The distal end of the tibia was fixed in all directions.^36^

### 2.4 Material properties

During the healing process, the MSCs within the fracture callus differentiate into fibroblasts, chondrocytes and osteoblasts.^46^ All materials were assumed to be isotropic. The material properties of the different tissues used in the bone fracture repair and bone remodelling algorithm are listed in Table 1.

**TABLE 1:** Material properties for bone plate and bone

	Elastic modulus (MPa)	Permeability (m^4^/Ns)	Poisson's ratio	Initial porosity	Solid bulk modulus (MPa)	Fluid modulus (MPa)
Cortical bone	17,000^28^	1E−17^28^	0.37^28^	0.04^28^	2300^2^	2300^2^
Marrow	0.0247^47^	1E−14^28^	0.167^28^	0.8^28^	2300^2^	2300^2^
Granulation tissue	0.001–2^2^, ^28^	1E−14^28^	0.167^28^	0.8^28^	2300^2^	2300^2^
Fibrous tissue	0.2–5^28^	1E−14^28^	0.167^28^	0.8^28^	2300^2^	2300^2^
Cartilage	5–500^28^	5E−15^28^	0.167^28^	0.8^28^	2300^2^	13,920^2^
Immature bone	500–1000^28^	1E−13^28^	0.3^28^	0.8^28^	2300^2^	13,920^2^
Intermediate bone	1000–2000^28^	3.7E−13^28^	0.3^28^	0.8^28^	2300^2^	13,920^2^
Mature bone	2000–6000^28^	3.7E−13^28^	0.3^28^	0.8^28^	2300^2^	13,920^2^
Titanium (Grade 2)	110,000^48^	‐	‐	‐	‐	‐

### 2.5 Fracture healing model calibration

**FIGURE 7:**
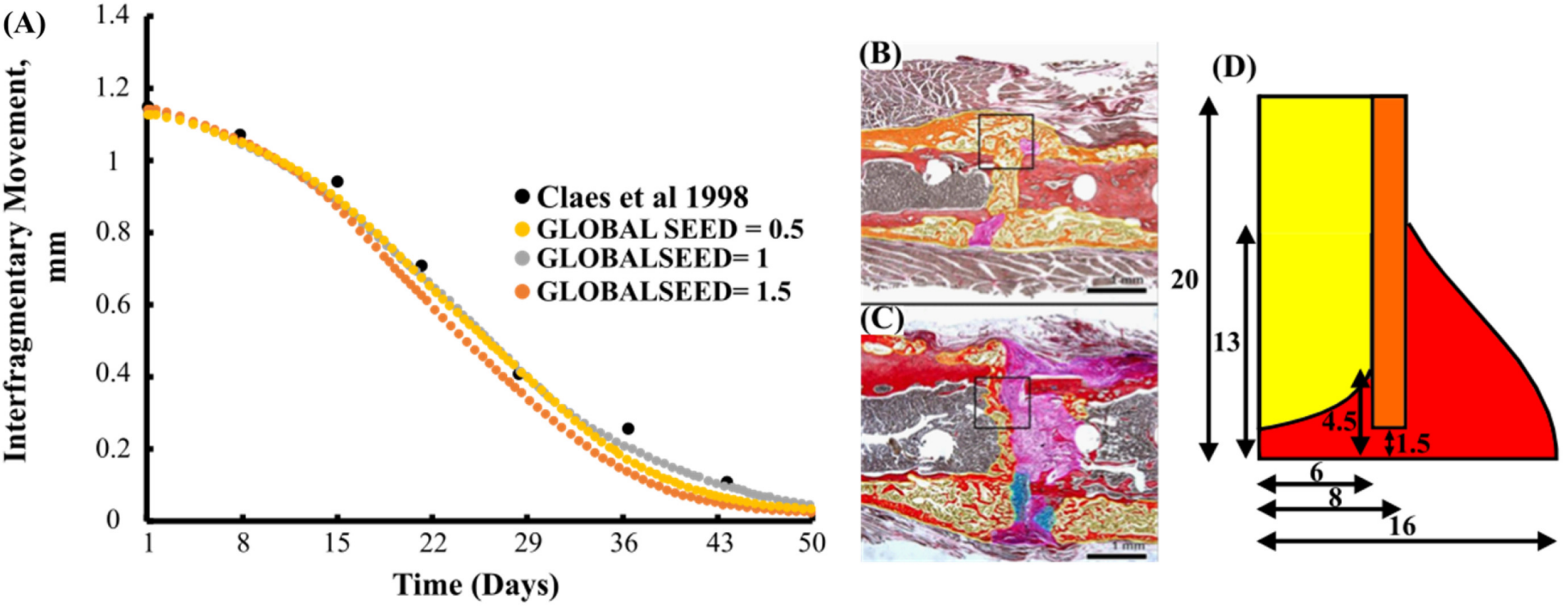
(A) Predicted IFM by FE‐model versus healing time of an osteotomy of a sheep. (B) Histology section of a well‐healed mouse at day 26. (C) Histology section of a mouse with mixed healing at day 26. Stained with Hall's and Brunt's Quadruple (HBQ) stain and false‐coloured. Blue = cartilage, yellow = trabecular bone, purple = fibrous/amorphous tissue, red = cortical bone, black/white = bone marrow.^41^(D) Geometrical dimensions of one quarter of FE‐model of the callus region used fracture healing model calibration^3^

A 3 mm mid‐diaphyseal osteotomy was induced in the metatarsus of a sheep and stabilised with a unilateral external fixator. The geometry used for fracture healing model calibration (see Figure 7D) was adapted from Claes et al.^3^ to create a three‐dimensional model which included bone marrow. An initial displacement of 1.13 mm was induced through the application of a 500 N force. Calibration of the model produced the following model parameters: cell differentiation rates $F_{\mathrm{mb}}$ = 0.15 day^−1^, $F_{\mathrm{ib}}$ = 0.15 day^−1^, $F_{c}$ = 0.3 day^−1^, $F_{f}$ = 0.02 day^−1^, tissue production rates $Q_{\mathrm{mb}}$ = 0.1 day^−1^, $Q_{\mathrm{ib}}$ = 0.1 day^−1^, $Q_{c}$ = 0.2 day^−1^ and $Q_{f}$ = 0.12 day^−1^. Using these parameters, the model was accurately able to reproduce the interfragmentary strain obtained by Claes et al.^3^ Here, the model does not predict the formation of a complete bony bridge after 50 days in the osteotomy gap, leaving a portion of the callus filled with fibrous and cartilage tissue, which corresponds with the experiments carried out by Claes et al.^3^

Mesh sensitivity for fracture healing was carried out using a global seed (**GS**) of 1.5, 1 and 0.5, which resulted in 4256, 12,547 and 91,256 elements. Element size had minimal on the healing outcome as shown in Figure 7A. For all simulations, a global seed of 1.5 was implemented.

## 3 RESULTS

### 3.1 Fracture repair‐effect of loading conditions (non‐plated)

**FIGURE 8:**
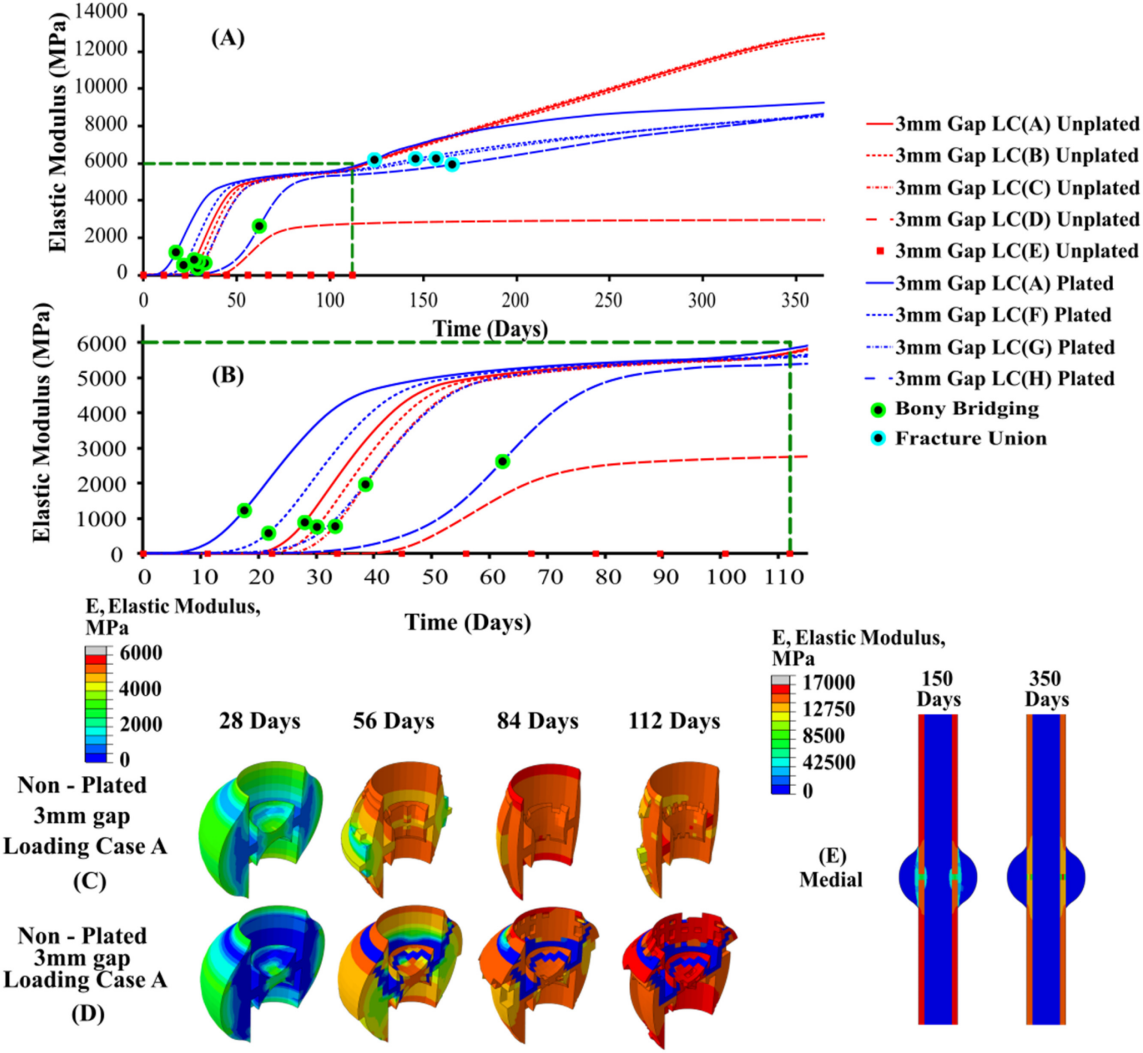
Elastic modulus of fractured tibia over a 1‐year time period with a constant fracture gap and different loading conditions; (A) fracture callus healing performance over 1 year; (B) fracture callus healing performance over 112 days; (C–E) contour plots of fracture callus; (F) SED/micro‐damage based remodelling

Figure 8A,B shows the predicted mean elastic modulus in the callus focus region (e.g., between both cortex ends) over the course of 365 days, based on the coupled fracture healing and bone remodelling algorithms, under a range of different loading conditions. For the non‐plated 3 mm fracture gap, the rate of healing was reduced when higher loading regimes were applied. Here, it is shown that the formation of a bony bridge occurred at 16.6 days for LC(A), which was the smallest loading regime, resulting in the fastest rate of healing with fracture union achieved at 120 days. For increasing loading magnitudes, the time to form a bony bridge and fracture union was progressively longer. For excessive loading conditions in LC(D) and LC(E), there is little or no increase in elastic modulus over the first 120 days. The higher loads inhibited the formation of a bony bridge, preventing the stabilisation of fracture callus region and resulted in the formation of a non‐union.

The coupled fracture repair framework only allows the callus to enter SED based remodelling once a fracture union had formed between the cortical ends. During remodelling, the SED/microdamage based framework caused the outer and inner callus to be predominately negatively remodelled and reabsorbed. Meanwhile, the callus focus region underwent positive remodelling as shown in Figure 8E. The undamaged cortical bone initially negatively remodelled under the reduced physiological load, however as loading returns to normal levels, the undamaged cortical bone was positively remodelled and returned to homeostatic levels (see Figure 8E).

### 3.2 Fracture repair and remodelling‐effect of fracture gap

**FIGURE 9:**
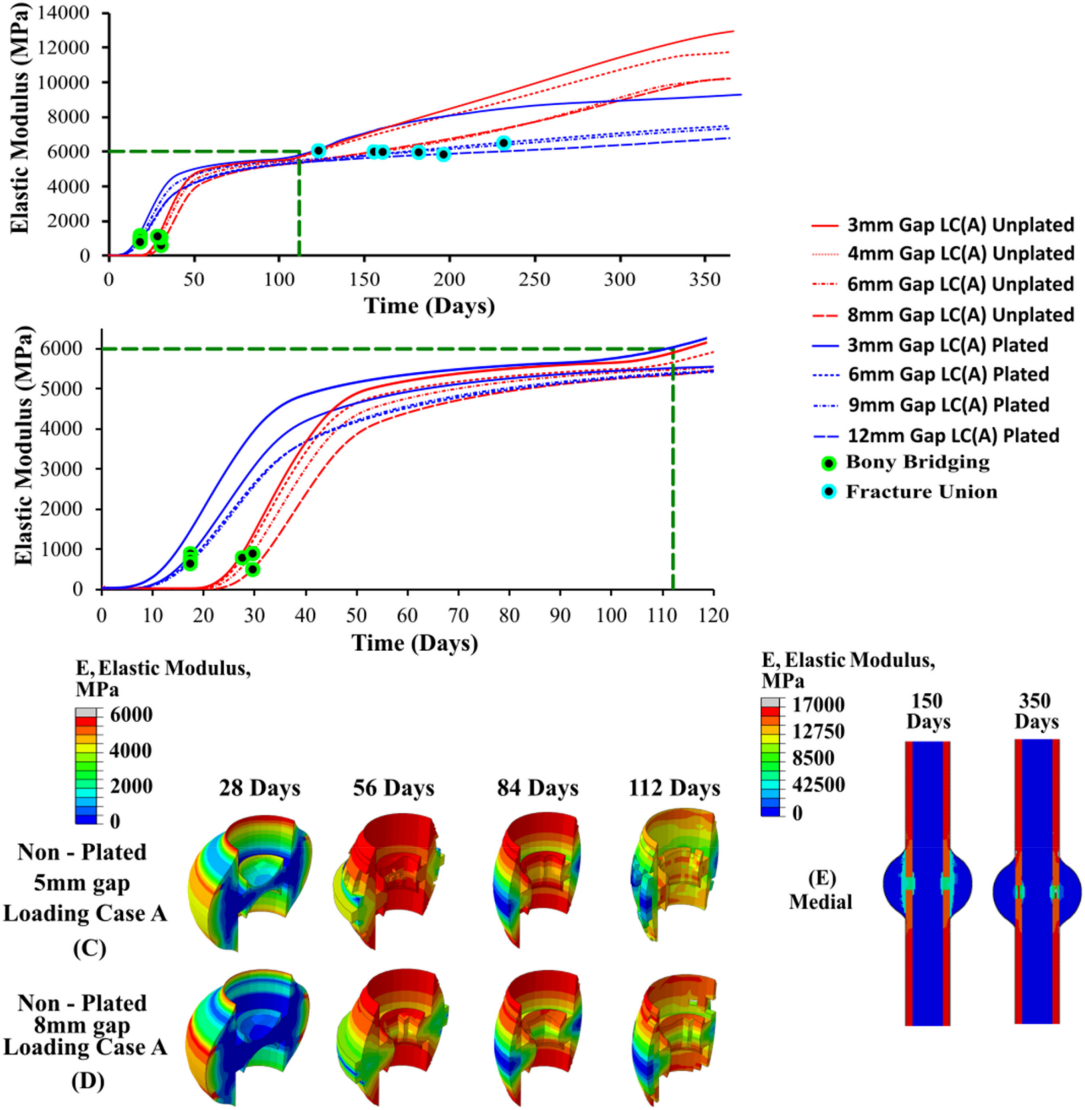
Elastic modulus of fractured tibia over a 1‐year time period with a constant loading condition and different fracture gaps; (A) fracture callus healing performance over 1 year; (B) fracture callus healing performance over 112 days; (C–E) contour plots of fracture callus; (F) SED/micro‐damage based remodelling

Figure 9 shows the predicted elastic modulus in the callus over the 365 days, based on the coupled fracture repair framework for loading condition LC(A) and fracture gaps of 3, 4, 6 and 8 mm. Here, the fracture healing performance was directly related to the fracture gap size, with the 3 mm showing the fastest rate of healing. Increasing the fracture gap size slowed the rate of fracture healing, as there was a slower infiltration of cells to fracture site, in addition to an increased biophysical stimulus due to geometry. Remodelling proceeded like before, with the inner and outer callus undergoing negative remodelling, while the callus focus region underwent positive remodelling. Where a union is successfully formed, the elastic modulus of the remodelled tissue is very similar across all cases after 365 days.

### 3.3 Fracture repair—Effect of plating

**FIGURE 10:**
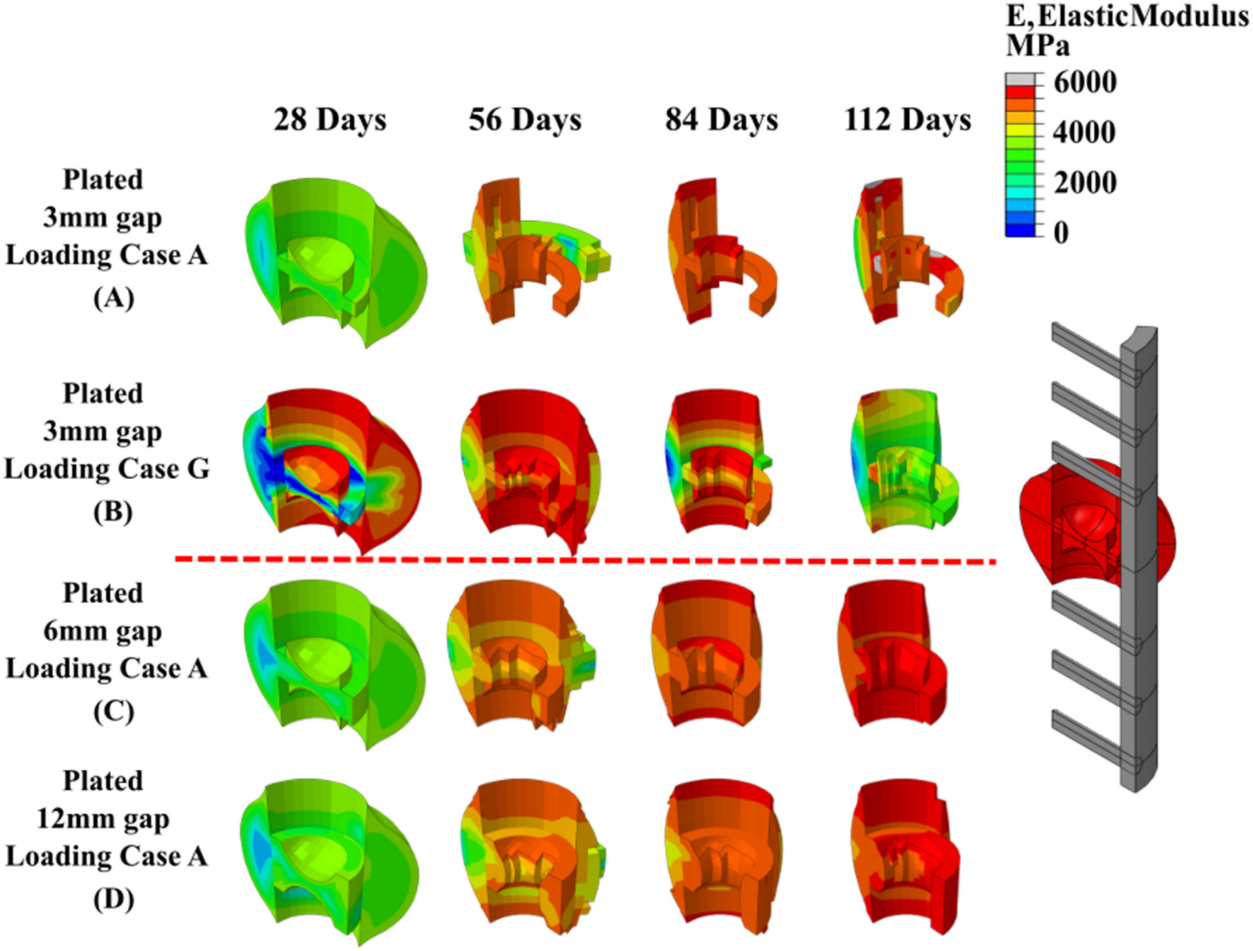
Contour plots of plated fracture callus; (A–C) fracture callus with a constant fracture gap and different conditions; (D–E) fracture callus with a constant loading condition and different fracture gaps; (E) wireframe representation of callus and plate positions

The effects of the implantation of a titanium plate on fracture healing and remodelling was investigated for LC(A), LC(F)–LC(H). Figure 8B shows that the predicted elastic modulus in the callus during the 365 days of repair for these loading conditions, while Figure 9B shows the predicted elastic modulus during repair for different fracture gap sizes for LC(A). Figure 8B shows that the introduction of a titanium plate allowed the tibia to undergo successful healing at much higher loading conditions, compared with the un‐plated versions. Non‐union occurred for the un‐plated case under LC(C) (0.33 MPa), while the plated version successfully healed up to LC(H) (6.6 MPa), which had substantially higher magnitudes applied (see Figure 6). The introduction of a stiff plated increased the overall rate of fracture repair, which is clear when comparing the plated/un‐plated models utilising LC(A). Figure 10 shows the healing profile of the callus itself, where the presence of the plate affects the spatial progression of healing. Tissues positioned closer to the plate stiffened and was reabsorbed quicker than tissue positioned further away for the plate. While the plated cases showed faster healing rates, their performance was substantially different once they entered the remodelling phase, with all plated cases remodelling to a much lower elastic modulus than any of the un‐plated loading cases (see Figure 8). The introduction of a titanium plate allowed for successful fracture healing to occur at much larger fracture gaps, although with a delayed healing performance with increasing fracture gaps **(**Figure 9).

**FIGURE 11:**
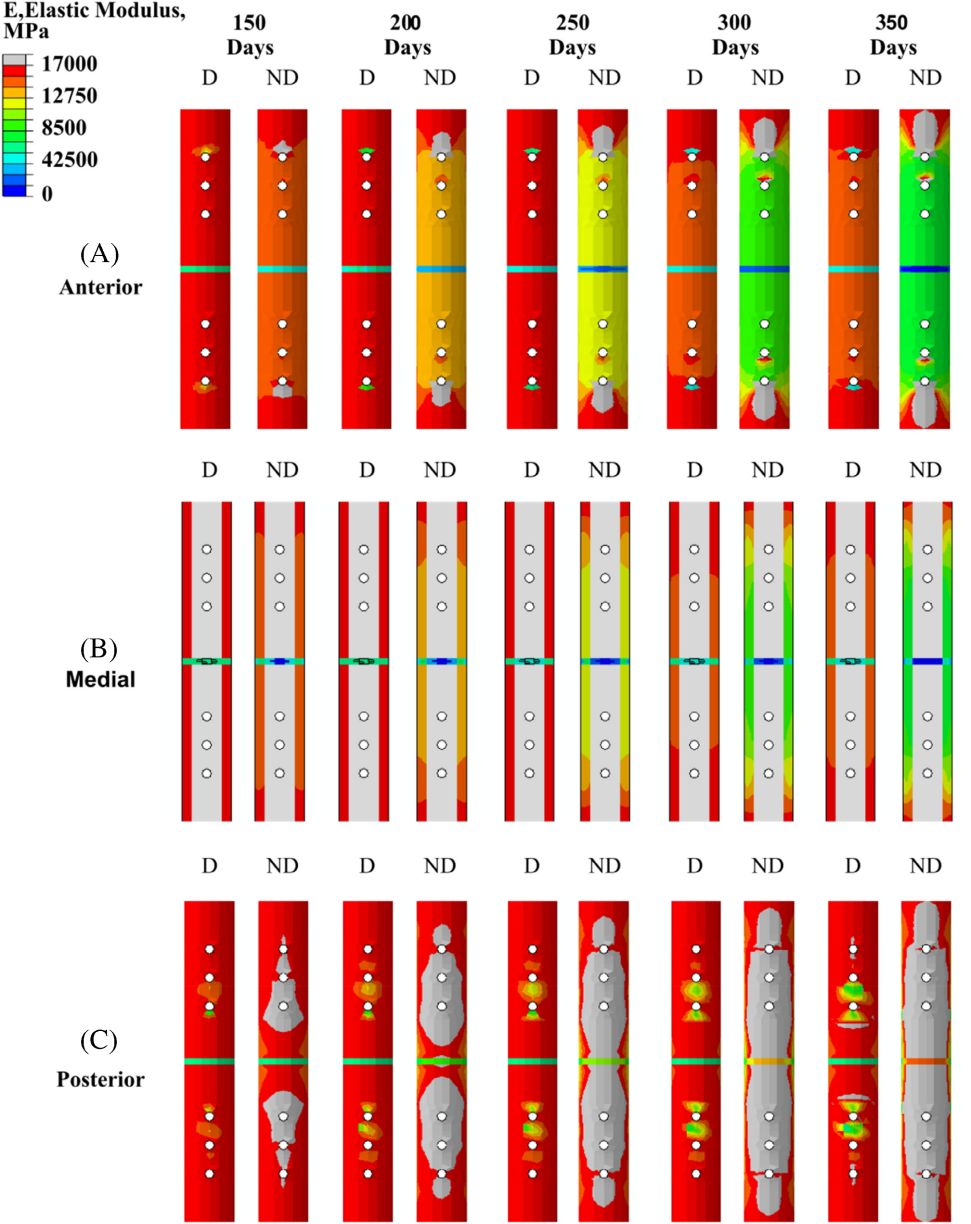
Elastic modulus within cortical bone and fracture callus focus for plated tibia with 3 mm fracture gap under LC(A) at 150, 200, 250, 300 and 350 days. Simulations were ran with damage (D) and with the no damage (ND)

In Figure 11, posterior, medial and anterior cuts of the tibia at 150, 200, 250, 300 and 350 days show the stress shielding and damaged‐based remodelling that occurs due to introduction of the plate. The remodelling framework considered cases with damage (D) and with no damage (ND). The highest degree of stress shielding occurred anteriorly (Figure 11A), while the least amount of stress shielding occurred posteriorly as seen in (Figure 11C). Stress concentrations were created locally, mainly in the interfacial regions between the titanium pins and cortical bone. Models containing damage (D) led to damage‐based reabsorption around the pins, while the models without damage (ND) the high stresses resulted in positive SED remodelling around the pins. A significantly higher degree of stress shielding around the plate was observed for the models containing ‘no damage’ than the models containing ‘damage’ (Figure 11A).

## 4 DISCUSSION

In this study, a coupled biphasic model for bone fracture repair was developed that considers both fracture healing and remodelling phases of repair to investigate the performance of a fractured tibia in both non‐plated and plated conditions. It was found that introduction of a titanium plate allowed the tibia to undergo successful healing at higher loading conditions and fracture gaps, compared to the non‐plated versions. While these plated cases showed faster rates of repair in the healing phase, their performance was substantially different once they entered the remodelling phase, with all plated cases remodelling to a much lower elastic modulus than any of the non‐plated loading cases, with the coupled modelling framework predicting substantial regions of stress shielding. This framework is one of the few implementations of coupled fracture healing and remodelling algorithms and included several innovative approaches to smoothing, time‐averaging and time incrementation in its implementation, thereby avoiding any unwanted abrupt changes in tissue phenotypes. Previous papers^6^ implemented time averaging within a static time increment $\left(\mathrm{dt}=1\right)$, therefore the updated elastic modulus was always updated over 10 increments (see Equation (19)). No study has implemented time averaging with a variable time step allowing for greater flexibility, control and convergence when modelling.

This coupled model provides a better representation of tissue development in the fracture callus and cortical bone when compared with fracture healing alone and provides novel platform to investigate the long‐term performance of orthopaedic fixation devices. This would include the more effective design of permanent fixators and provide an opportunity to optimise both spatial and temporal behaviour of bioabsorbable implants to limit unwanted effects of stress shielding.

The coupled fracture repair model was shown to provide very good correlation to animal experiments carried out by Claes et al.,^49^ in terms of both predicted inter‐fragmentary strain (see Figure 7) and the time sequence of the repair.^41^ During the fracture healing phase, the simulations generally predicted the formation of the cartilaginous callus 2–3 weeks from the start of the healing process.^2^ The extremities of the fracture callus experience lower biphasic stimulus and stiffen more quickly. The bony bridge provides mechanical support lowering the biphasic stimuli at the fracture site, allowing for bone formation to occur in other regions such as near the bone marrow and fracture gap. The formation of a complete “bony bridge” was a pivotal aspect in achieving successful healing (see Figures 8, 9 and 10), and the coupled fracture repair model used this feature as the criteria that determined whether the bone remodelling phase would be initiated (the time at which this occurred was not prescribed a priori). Upon successful fracture healing, the non‐plated models remodelled positively, tending towards the original density of cortical bone (ρ = 1.649 g/cm^3^, E = 17,000 MPa). This was largely driven by the fact that a homeostatic load of 6.6 MPa was assumed to be acting on the tibia after day 112 for all conditions, resulting in similar bone densities for the non‐plated models. Due to the geometry of the non‐plated models, they were remodelled in a symmetric manner, with the inner and outer callus undergoing negative remodelling, while the callus focus region underwent positive remodelling (see Figure 8). During all transitions, the coupled fracture repair algorithm was implemented in such a way that abrupt changes in tissue modulus due to local stimuli were avoided. This was achieved through the implementation of several time incrementation controls and smoothing techniques (see Section 2). For the latter, the change in elastic modulus due to the biphasic stimulus and SED were time averaged over 10 days. Therefore, a change in the elastic modulus of an element was due to its stimulus history, opposed to a single time instance as was commonly employed in other studies.^50^, ^51^, ^52^

The implantation of a titanium plate played a critical role in healing and remodelling phases of bone repair, creating an uneven stress distribution across the fracture callus that resulted in substantially different healing outcomes between the plated and non‐plated models. During fracture healing, regions directly adjacent to the plate experience lower biophysical stimuli than regions on the opposite side. Based on the mechano‐regulatory framework, these elements transition through tissue phenotypes more quickly, leading to local stiffening, with some regions even being absorbed due to the lack of a biophysical stimulus during fracture healing (See Figure 10). Upon entering the remodelling phase, most of the stress is distributed through the implant, as it has a stiffness of ~6.5 times greater than cortical bone, thereby shielding the adjacent region. The proximity of the surrounding tissue to the titanium plate heavily influenced the degree of stress‐shielding. The highest degree of stress shielding occurred anteriorly, while the least amount of stress shielding occurred posteriorly (see Figure 11). This subsequently resulted in the anterior portion of the ‘callus focus’ region to negatively remodel, while the posterior portion remodelled positively producing a cortical shell. Most of the bone loss around the titanium plate occurred between 0 and 6 months for the ‘damage’ models with a significant reduction in bone loss following the 6‐months, matching experimental tests of an implanted titanium stem carried by Jaffe and Scott.^53^ Interestingly, during the remodelling phases, the plated models also predicted further resorption in stress concentration regions where screws were fastened to the bone. The stress concentrations were generated due to (i) the dissimilar stiffness of titanium (E_T_ = 110GPa) and cortical bone (E_c_ = 17GPa) and (ii) the geometry of hole generated by the titanium pin. In this study, the role of damage‐based reabsorption was during remodelling was considered. Within the ‘no‐damage’ models, the high stresses generated around the pins led to high levels of positive remodelling. This subsequently led to higher levels of stress being redistributed through the titanium plate increasing the stress‐shielding effect. On the contrary, when damage was included in these models, these high stresses led to damage‐based reabsorption in these regions (which is a completely separate effect to stress‐shielding). Interestingly, the effect of this damage‐based resorption actually redistributed stress away from the pins and subsequently the plate, thereby substantially reducing the stress shielding effect. Damage‐based reabsorption occurs much more rapidly than negative SED remodelling, which may cause implant failure as the interfacial bone around the pins is reabsorbed, resulting in bone plate slippage.

The healing performance for both the plated and non‐plated simulation were directly impacted by the loading conditions (Figures 8 and 10) and fracture gap size (see Figure 9). For the simulations in which the load was altered the fracture gap remained constant at 3 mm, while for all different fracture gap sizes, the base loading condition LC(A) was used. Under different loading regimes (in the first 28 days), it was found that the time at which the ‘bony bridge’ formed was different in both plated and non‐plated scenarios. The plated models were able to successfully heal at much higher loads (e.g., LC(H) when compared with the non‐plated models (e.g., LC(C)). An increase in fracture gap size decreased the healing performance for both plated and non‐plated simulations. Larger fracture gaps increased the biphasic stimulus induced within the fracture region for the same loading conditions. For all fracture gaps the diffusion coefficient remained constant at 0.3 mm/day. This, in turn, influenced the time taken for the callus focus region to become confluent in MSCs, and thus slowed the healing response. The simulated results obtained here mimic that of experimental results seen in a rat model conducted by Meeson et al.,^54^ whereby a femoral osteotomy of different sizes (1, 1.5 and 2 mm) resulted in delayed healing over a 5 week period.

The prediction of long‐term outcomes of bone fracture repair represents an extremely challenging problem that involves highly complex interaction between physical, chemical and biological processes taking place at the fracture callus. This implies that the coupled healing and remodelling framework presented here is subject to several limitations. Both the fracture healing and remodelling algorithms rely on sets of phenomenological rules to drive cellular activity, according to a series of thresholds that dictate tissue formation at different stages of the process. For these, threshold parameters have been chosen based on existing experimental and computational literature across both algorithm types^26^ and this coupled framework uses a novel numerical implementation, whereby abrupt changes between tissue phenotype is avoided through suitable smoothing, time‐averaging and automatic time incrementation strategies. In validating the coupled framework, the model predictions showed excellent agreement with inter‐fragmentary movement (IFM) from an animal experiment, with differences below 0.1 mm seen throughout the healing phase. The model also produced typical patterns and appropriate time sequences that have been observed in both bone healing and bone remodelling phases across animal and human studies.^3^, ^41^ Therefore, this coupled model provides a suitable platform to investigate bone fracture repair and the long‐term performance of orthopaedic fixation devices.

In order to simulate fracture healing and bone remodelling effectively several simplifications were made regarding model geometry and boundary conditions. The idealised geometry allowed for a great reduction in the number of elements, reducing computational cost. Additionally loading was modelled as one loading cycle per day, therefore ignoring the accumulative effect of repetitive cyclic loading. Both simplifications would prevent the model outlined in this paper capturing the actual stress distribution of a fractured tibia in vivo. Additional limitations include a mismatch include limited experimental data, and a lack of experimental case studies for clinical applications.^55^ Ideally mechanoregulatory models should be patient specific: the geometry, boundary conditions and model parameters.

The results outlined within this paper provide a valuable resource into orthopaedic device design. Numerous studies when considering bone fracture repair only consider plating for the first 3–6 months, not considering the considering the long‐term effects of permanent platting. The algorithm outlined within this paper provides a quantifiable output to analyse the effects platting has on short‐term bone fracture repair and long‐term bone remodelling providing a more realistic result. Therefore, allowing for greater scope when optimising implanted orthopaedic device design.

## 5 CONCLUSION

In this study, a coupled biphasic model for bone fracture repair was developed that considers both fracture healing and remodelling phases of repair and investigates the performance of a fractured tibia in both non‐plated and plated conditions. It was found that introduction of a titanium plate allowed the tibia to undergo successful healing at higher loading conditions and fracture gaps, compared to the non‐plated versions, but resulted in substantial regions of stress shielding at later stages of repair. It was found healing time was increased when increases in either loading conditions (Figures 8 and 10) or fracture gap size (see Figure 9) were considered. This model representation of tissue development in the fracture callus and cortical bone when compared with fracture healing alone provides novel platform to investigate the long‐term performance of orthopaedic fixation devices. This approach could be used to optimise the spatial and temporal load‐bearing performance of bioabsorbable fixation devices, to enhance repair outcomes.

## FUNDING INFORMATION

Funding support was provided by the Irish Research Council (IRC) Government of Ireland Postgraduate Scholarship (GOIPG/2017/2102). The project also recieved funding from the European Union's Horizon 2020 research and innovation programme under grant agreement No 813869. This publication reflects only the authors' view and the REA is not responsible for any use that may be made of the information it contains.

## CONFLICT OF INTEREST

The authors declare no conflict of interest(s) in the work presented.

## Data Availability

The model is implemented in the Abaqus/Standard finite element framework, with several user‐defined subroutines written to predict fracture healing and bone remodelling. A set of example files have been included in the GitHub repository https://github.com/tedvaughan/Fracture-Healing-and-Remodelling-Model-.git
